# Neurocognitive and somatic stabilization in pediatric patients with severe Mucopolysaccharidosis Type I after 52 weeks of intravenous brain-penetrating insulin receptor antibody-iduronidase fusion protein (*valanafusp alpha*): an open label phase 1-2 trial

**DOI:** 10.1186/s13023-018-0849-8

**Published:** 2018-07-05

**Authors:** Roberto Giugliani, Luciana Giugliani, Fabiano de Oliveira Poswar, Karina Carvalho Donis, Amauri Dalla Corte, Mathias Schmidt, Ruben J. Boado, Igor Nestrasil, Carol Nguyen, Steven Chen, William M. Pardridge

**Affiliations:** 1 Hospital de Clínicas de Porto Alegre and UFRGS, Porto Alegre, Brazil; 2 ArmaGen, Inc., 26679 Agoura Road, Calabasas, CA USA; 3 Department of Pediatrics and Adolescent Health, University of Minnesota, 717 Delaware St. SE, Minneapolis, MN 55414 USA; 4 Department of Radiology, Duke University, Durham, NC 27710 USA

**Keywords:** Mucopolysaccharidosis Type I, Iduronidase, Blood-brain barrier, Insulin receptor, Open label clinical trial, Safety, Efficacy

## Abstract

**Background:**

Mucopolysaccharidosis (MPS) Type I (MPSI) is caused by mutations in the gene encoding the lysosomal enzyme, α-L-iduronidase (IDUA), and a majority of patients present with severe neurodegeneration and cognitive impairment. Recombinant IDUA does not cross the blood-brain barrier (BBB). To enable BBB transport, IDUA was re-engineered as an IgG-IDUA fusion protein, valanafusp alpha, where the IgG domain targets the BBB human insulin receptor to enable transport of the enzyme into the brain. We report the results of a 52-week clinical trial on the safety and efficacy of valanafusp alpha in pediatric MPSI patients with cognitive impairment. In the phase I trial, 6 adults with attenuated MPSI were administered 0.3, 1, and 3 mg/kg doses of valanafusp alpha by intravenous (IV) infusion. In the phase II trial, 11 pediatric subjects, 2-15 years of age, were treated for 52 weeks with weekly IV infusions of valanafusp alpha at 1, 3, or 6 mg/kg. Assessments of adverse events, cognitive stabilization, and somatic stabilization were made. Outcomes at 52 weeks were compared to baseline.

**Results:**

Drug related adverse events included infusion related reactions, with an incidence of 1.7%, and transient hypoglycemia, with an incidence of 6.4%. The pediatric subjects had CNS involvement with a mean enrollment Development Quotient (DQ) of 36.1±7.1. The DQ, and the cortical grey matter volume of brain, were stabilized by valanafusp alpha treatment. Somatic manifestations were stabilized, or improved, based on urinary glycosaminoglycan levels, hepatic and spleen volumes, and shoulder range of motion.

**Conclusion:**

Clinical evidence of the cognitive and somatic stabilization indicates that valanafusp alpha is transported into both the CNS and into peripheral organs due to its dual targeting mechanism via the insulin receptor and the mannose 6-phosphate receptor. This novel fusion protein offers a pharmacologic approach to the stabilization of cognitive function in MPSI.

**Trial registration:**

Clinical Trials.Gov, NCT03053089. Retrospectively registered 9 February, 2017; Clinical Trials.Gov, NCT03071341. Registered 6 March, 2017.

## Background

Mucopolysaccharidosis (MPS) Type I (MPSI) can present as severe MPSI (Hurler syndrome) or attenuated MPSI (Hurler-Scheie, or Scheie syndrome), and is caused by mutations in the gene encoding the lysosomal enzyme α-L-iduronidase (IDUA) [1]. Recombinant IDUA (laronidase, Aldurazyme®) was developed as intravenous (IV) Enzyme Replacement Therapy (ERT) for the treatment of MPSI [1, 2]. Patients with Hurler Syndrome, which constitutes about half of the patients [3], and the more severely affected Hurler-Scheie patients, exhibit neurodegeneration and cognitive impairment. Laronidase has no impact on cognitive decline in MPSI [4], because the enzyme does not cross the blood-brain barrier (BBB) [5]. Hematopoietic stem cell transplant (HSCT) is believed to stabilize the neuronal degeneration in MPSI, particularly if the HSCT is performed before the age of 16 months [6]. In addition to the morbidity and mortality associated with HCST, there remains a significant portion of MPSI patients that are permanently cognitively impaired following HSCT [6, 7].

A BBB-penetrating form of IDUA would allow for a non-invasive therapeutic option to treat the central nervous system (CNS) manifestations in MPSI. The IDUA enzyme can be made transportable through the BBB following the re-engineering of the lysosomal enzyme as an IgG-IDUA fusion protein, where the IgG domain is a receptor-specific monoclonal antibody (MAb) that targets an endogenous BBB receptor transporter, in this case the human insulin receptor (HIR). The IgG-IDUA fusion protein was formed by fusion of the human IDUA enzyme, without the enzyme signal peptide, to the carboxyl terminus of each heavy chain of a genetically engineered chimeric HIRMAb [8]. The HIRMAb domain of the HIRMAb-IDUA fusion protein triggers receptor mediated transport of the fusion protein into brain via the endogenous BBB insulin receptor, and acts as a molecular Trojan horse to ferry into brain the IDUA fused to the IgG domain [5, 8]. The HIRMAb-IDUA fusion protein is alternatively designated as AGT-181 [9], or as the international non-proprietary (rINN) name of valanafusp alpha. A surrogate fusion protein reduces lysosomal inclusion bodies in the brain of the MPSI mouse following chronic IV administration [10].

The IDUA domain of valanafusp alpha incorporates mannose 6-phosphate (M6P) [5], which allows uptake also into somatic tissues via the M6P receptor (M6PR), similar to recombinant IDUA. Whole body autoradiography in primates shows a comparable biodistribution in peripheral organs for laronidase and valanafusp alpha [5]. However, the M6PR is not expressed at the human BBB, and laronidase does not penetrate the monkey brain [5]. Conversely, there is global penetration of the CNS by valanafusp alpha in the primate owing to BBB transport of the fusion protein via the endogenous insulin receptor [5]. The dual receptor targeting of the HIRMAb-IDUA fusion protein provides the rationale for reversal of lysosomal inclusions in both somatic and CNS tissues following chronic IV treatment of MPSI subjects with valanafusp alpha.

The present study reports on a phase I-II clinical trial of the treatment of MPSI adults and children with valanafusp alpha. After a single dose-escalation phase I trial in 6 adult MPSI subjects, a phase II trial in 11 pediatric MPSI patients was performed over 52 weeks of chronic weekly IV infusions of valanafusp alpha. The plasma pharmacokinetics of valanafusp alpha in adults and children with MPSI has been described previously [11]. In the present investigation, the safety, tolerability, and stabilization of somatic and cognitive function by valanafusp alpha are evaluated in MPSI children with severe mental retardation. This study is the first to address the response to drug therapy of both cognitive and somatic outcomes in MPSI patients with severe cognitive impairment. None of the patients enrolled in this trial would be able to receive HSCT. This investigation is also the first human clinical trial of a BBB molecular Trojan horse fusion protein.

## Methods

### Study design and patients

**Fig. 1:**
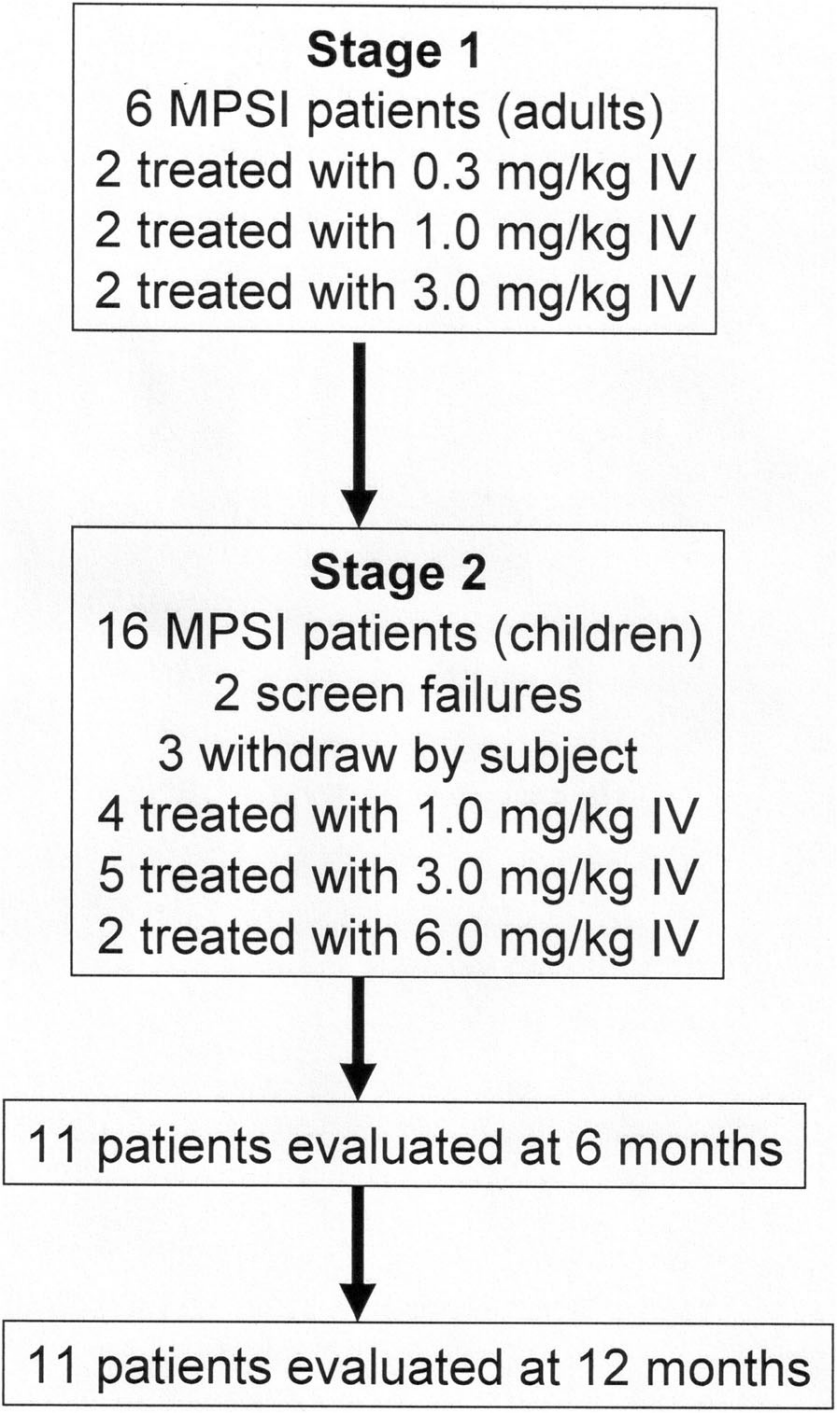
Trial design

In Stage 1, a total of 6 adult Scheie MPSI subjects were enrolled between October, 2015 through January, 2016, and treated with a single IV infusion of 0.3, 1, and 3 mg/kg of valanafusp alpha (Fig. 1). In Stage 1, all subjects were female and all had a negative pregnancy test. The fusion protein was infused IV over 3-4 hours in normal saline with 5% dextrose (D5NS). After evaluation of safety data accrued over a 4-week period, a total of 16 pediatric Hurler or Hurler-Scheie MPSI subjects were identified for enrollment in the trial between March, 2016 through January, 2017. Two patients failed screening tests, and 3 patients withdrew early from the study owing to travel constraints. The remaining 11 pediatric subjects, all males, remained in the 6-month study followed by a 6-month extension, with all 11 subjects undergoing treatment for 12 months. Of these 11 patients, 9 had been previously on laronidase ERT, 2 patients had not previously been treated with ERT, and 1 patient had undergone a failed bone marrow transplant prior to enrollment in the study (Table 2). All patients were confirmed to have MPSI by genotype and low leukocyte IDUA enzyme activity.

**Table 1:** Stage 1 Scheie patients

patient	Age (years)	Genotype	
		Allele 1	Allele 2
101	28	p.R89Q	p.W402X
102	37	p.Q70X	p.D445del
103	19	IVS7+1G>A	p.X654R
104	31	c.590-6ins4	p.Y343X
105	30	p.R383H	p.W402X
106	24	p.R383H	p.W402X

**Table 2:** Stage 2 Hurler and Hurler-Scheie patients

patient	Type	Dx age (y)	Yrs on ERT	Height (cm)	Weight (kg)	Enroll. Age (y)	ID (mg/kg)	Genotype	
								Allele 1	Allele 2
202	H	0.7	8	101	19.6	9.0	1	p.W402X	p.W402X
203	H-S	4.9	1.7	104	19.1	6.1	3	p.R621X	het IVS9-12-4delCAGGCCCCG
204	H-S	5.0	9	144	37.1	14.8	1	p.R48del	p.P533R
205	H	1.1	1.5	112	24.7	5.0	1	c.494-1G&gt;A	c.494-1G&gt;A
206^a^	H	1.8	1	81	11.6	3.1	1	p.W402X	p.W402X
207	H	2.6	3	95	18.8	5.9	3	p.Q70X	p.W402X
211	H	2.4	0	78	11.4	2∙5	3	p.G208D	p.G208D
213	H-S	3.7	12	143	38.5	15.7	3	p.R383H	p.W402X
214	H	1.8	0	85	13.0	2.0	3	p.W402Term	p.W402Term
215	H	3.6	5.6	137	39.6	12.1	6	p.P533R	p.G208D
216	H	0.7	5.6	118	26.4	7.8	6	p.G208D	p.P533R

^a^patient had failed bone marrow transplant 8 months prior to enrollment

The study drug, valanafusp alpha, also called AGT-181 [9], had an IDUA enzyme specific activity of 1390-1668 units/ug protein, where 1 unit = 1 nmol/hr, and bound with high affinity to the recombinant HIR extracellular domain with an ED50 of 0.18-0.36 nM.

### Procedures and Assessments

The pharmacokinetics (PK) of plasma clearance of 6 adults and 13 children with MPSI were determined in the first week of treatment and these PK results have been recently reported [11]. This PK analysis was performed on plasma IDUA enzyme activity following the IV infusion of valanafusp alpha. For stage 1, blood samples and urinalysis were tested weekly for 4 weeks. For stage 2, blood samples and urine were taken at 0, 4, 8, 13, 17, 22, 26, 30, 34, 42, and 52-54 weeks for 19 tests of clinical chemistry, 15 hematology tests, and urinalysis. Thyroid function (free T4 and TSH), and cervical spine MRI, were evaluated at screening for all subjects. Cerebrospinal fluid (CSF) pressure (cm water) was measured by lumbar puncture in the lateral decubitus position at baseline and at 26 weeks. Spot urine was collected for creatinine and glycosaminoglycans (GAGs) at 0, 4, 8, 13, 26, 30, 34, 42, and 46 weeks, and urinary GAGs (uGAGs) were reported as ug GAG per mg urinary creatinine. Left and right shoulder flexion and extension range of motion (ROM) were determined by goniometry at 0, 4, 13, and 26 weeks. Pulmonary function tests could not be performed owing to the age or cognitive impairment of the patients. Echocardiograms were performed on all subjects at 0 and 26 weeks. Electrocardiograms were performed on all subjects at 0, 4, 13, 26, 38, and 52 weeks. Brain, cervical spine, liver, and spleen MRI was performed on all subjects, under anesthesia, at 0, 13, 26, and 52 weeks with a Philips Achieva 1.5 Tesla MRI. Liver and spleen volumes were computed by manual segmentation using a region of interest approach and T2-weighted images with the OsiriX MD software. Brain volumetrics were quantified with the *FreeSurfer* Image Analysis Suite version 5.1 (Martinos Center, Harvard University, Boston, Massachusetts); the volume reported is for total gray matter volume, which is the sum of cerebral and cerebellar cortical and subcortical grey matter. The presence in patient serum of anti-drug antibodies (ADA) against valanafusp alpha was determined with a 2-site sandwich ELISA described previously [9, 12]. The capture agent is the valanafusp alpha and the detector agent is biotinylated valanafusp alpha. Owing to the bivalency of antibody binding, the ADAs bind both the capture and detector agents. This assay detects ADAs directed against either valanafusp alpha or laronidase, so any pre-existing ADAs against laronidase at baseline would be detected with the ADA assay. Blood glucose was measured in each patient during the 3-4 hour IV infusion to monitor hypoglycemia. Hypoglycemia in children is defined as a blood glucose <50 mg%, and serious hypoglycemia is defined as a blood glucose <40 mg% [13]. The lower limit of normal blood glucose in the assay is 60 mg%. For this study, grade 1, grade 2, and grade 3 hypoglycemia was defined as a blood glucose of 50-59 mg%, 40-49 mg%, and 30-39 mg%, respectively.

Cognitive testing was initially performed with the Vineland Adaptive Behavior Scales Second Edition (VABS-II). Those subjects with an Age Equivalent Score (AES) of <42 months were subsequently tested with the Bayley Scales of Infant and Toddler Development Third Edition (BSID-III), and those subjects with an AES>42 months were tested with the Kaufman Assessment Battery for Children Second Edition (KABC-II) [14]. The raw VABS, BSID, or KABC scores collected at the study site were converted to AES at NeuroCog Trials; the AES was divided by the subject’s chronologic age to compute the Development Quotient (DQ), which is an age-adjusted IQ score. The BSID-III test is comprised of 5 domains: cognitive, receptive communication, expressive communication, fine motor, and gross motor skills, and the cognitive domain was used as the primary measure of DQ. The KABC-II test is comprised of 8 domains: non-verbal index (NVI), conceptual, face recognition, story completion, triangles, block counting, pattern reasoning, and hand movements. The NVI, which represents a global or composite score of nonverbal subtests, was used as the primary measure of DQ. The VABS-II test examined the following 5 domains: overall, communication, daily living skills, socialization, and motor ability. The overall domain is computed from the communication, daily living skills, and socialization domains. Whereas the BSID and KABC DQ tests patient performance, the VABS test is a survey interview conducted by clinicians with the patient’s parent or guardian. All cognitive testing data was analyzed and maintained at NeuroCog Trials (Durham, NC).

Heparan sulfate (HS) and dermatan sulfate (DS) concentrations in cerebrospinal fluid (CSF) were measured at baseline and at 26 weeks. HS and DS levels were determined by liquid chromatography-mass spectrometry (LC-MS) at IAS, Inc. (Berkeley, CA), as described previously [15]. Control human pediatric CSF was obtained from BioIVT (Westbury, NY) for 10 subjects with an age of 6.4 ± 1.8 years (SEM).

### Statistical analysis

Statistical differences in liver and spleen volumes, as a percent of body weight (BW), at 52 weeks, relative to baseline, and differences in shoulder extension and flexion at 26 weeks, relative to baseline, were evaluated with the Paired T-Test and a two-tailed *P* value (GraphPad, La Jolla, CA).

### Ethics

All patients were treated and evaluated at the HCPA-Hospital das Clinical de Porto Alegre, Brazil. The clinical protocol was reviewed and approved by the local Institutional Review Board, the National Ethics Committee (CONEP) and the National Health Surveillance Agency (ANVISA) in Brazil. Written informed consent was read, understood, and signed by a parent or guardian before each patient enrolled in the study. All data was housed in a protected database managed by WCCT Global (Cypress, CA).

The clinical trial is recorded at Clinical Trials.Gov, with identifiers NCT03053089 for the phase 1-2 trial, and NCT03071341 for the open label extension study.

## Results

During Stage 1 of the trial, 6 female Scheie patients (Table 1), with a mean age of 28.2 ± 2.9 years (mean ± SE), were enrolled and all completed the study. These subjects underwent dose escalation from 0.3 to 3 mg/kg, and the only drug related adverse effect was a single plasma glucose of 39 mg% at the 3 mg/kg dose at the end of the infusion, which was resolved within 20 min. Serum chemistry and hematology was recorded weekly for 4 weeks following the single infusion with no abnormalities that were not observed at baseline.

During stage 2 of the trial, 4 patients were started on weekly IV infusions at 1 mg/kg, followed by the enrollment of 5 patients at the 3 mg/kg dose level, followed by 2 patients at the 6 mg/kg dose level. All 11 of these subjects continued through the 52-week duration of the study. The mean age of these patients was 7.6 ± 1.3 years (mean ± SE), and 9 subjects had been previously treated with laronidase ERT, with 2 ERT-naïve 2-year-old patients (Table 2). The mean body weight of the 11 pediatric subjects increased 18.2 ± 2.2% over 52 weeks from 23.6 ± 3.2 kg (SEM) at baseline to 28.2 ± 4.1 kg (SEM) at 52 weeks. The mean body height of the 11 pediatric subjects increased 5.7 ± 1.0% over 52 weeks from 109.0 ± 7.3 cm (SEM) at baseline to 114.6 ± 7.0 cm (SEM) at 52 weeks. The serum chemistry and hematology and urinalysis were stable over the course of 52 weeks of treatment. Any abnormalities observed were mild and also found at baseline. Many MPSI subjects had mild eosinophilia or monocytosis, mild anemia, and reduced serum creatinine, lactate dehydrogenase, or uric acid both at baseline and during the study. Mild ketonuria, proteinuria, and hematuria were observed at baseline and during the study (data not shown). The only drug related adverse events identified were infusion related reactions (IRR) and transient hypoglycemia (Table 3). Over the course of treatment of 11 subjects for 52 weeks, a total of 564 infusions were performed, which resulted in 10 IRRs, or an incidence of 1.7%; however, 6 of these IRRs were observed in a single patient, 214, a 2-year-old who entered the trial not previously on ERT. The IRRs in this patient were observed early in the course of treatment, were treated with corticosteroids, and were no longer observed by week 10 of treatment. Transient hypoglycemia was observed during 36 infusions or an incidence of 6.4%, and was resolved within 10-20 minutes of a snack or glucose sachet. The 6 mg/kg dose of fusion protein produced 67% of the transient hypoglycemic episodes (Table 3). Excluding the high dose of 6 mg/kg, the incidence of hypoglycemia at the 1-3 mg/kg doses was 2.1%. Of the 7 episodes of hypoglycemia observed at the 3 mg/kg dose, all were observed in a single patient, 213. The transient hypoglycemic episodes were 72% grade 1 (50-59 mg%), 19% grade 2 (40-49 mg%), and 8% grade 3 (30-39 mg%), and were treated with snacks or glucose sachets. The blood glucose during and after the infusions was measured over 3,000 times, and the mean glucose was 101±20 mg% (mean±SD), with a normal range of 60-100 mg%.

**Table 3:** Adverse events in stage 2

Adverse event	Number of adverse events in study		
	Infusion dose (mg/kg)		
	1	3	6
hypoglycemia	5	7	24
infusion reactions	0	10	0
URI	13	16	4
diarrhea	0	11	0
pyrexia	1	6	0
cough	3	4	0
sinusitis	4	2	0
otitis media	4	2	0
skin rash	1	4	0
back pain	3	0	2
impetigo	3	0	2

Adverse events listed were observed 5 or more times during course of 52-week study.

The primary DQ measure was the cognitive domain of the BSID-III, which was administered to 8 subjects, and the non-verbal index (NVI) domain of the KABC-II, which was administered to 3 subjects (Table 4). The mean DQ at enrollment of these 11 subjects was 36.1 ± 7.1 (SEM, Table 4). The DQ was stabilized over the course of 52 weeks and the difference in DQ at 52 weeks, relative to baseline, was -1.2 ± 2.8 (Table 4). The primary cognitive endpoint is also expressed as AES, and the mean change in AES at 52 weeks, relative to baseline was +5.5 ± 2.6 months (Table 4). The baseline DQ values measured with the VABS-II were somewhat higher, and the DQ was stabilized for each of the 5 domains of the VABS-II over the 52 weeks of treatment (Table 5). The mean AES of the 5 domains of the VABS-II increased between 7 and 21 months at 52 weeks relative to baseline (Table 5). The mean DQ for the 4 other domains of the BSID-III ranged from 13.8 ± 3.7 to 34.7 ± 7.7, and these DQ values were stabilized over the 52 weeks of treatment (Table 6). The mean AES of the 4 domains of the BSID-III shown in Table 6 increased between 0.1 and 4.3 months at 52 weeks relative to baseline (Table 6).

**Table 4:** AES and DQ and Change from Baseline of DQ at week 52 using BSID-III or KABC-II

patient	Test	AES (months)			DQ			
		Baseline	26 wk	52 wk	Baseline	26 wk	52 wk	52 wk-baseline
202	BSID-III cognition	0.5	9	6	0.45^a^	7.9	4.8	4.3
205		19	23	23	31.7	34.9	32.4	0.7
206		25	24	27	67.6	57.1	56.3	-11.3
207		10	16	na	14.1	20.8	na	7.7^b^
211		10	8	4∙7	33.3	20.0	10.2	-23.1
214		13	16	20	54.2	53.3	55.6	1.4
215		25	26	25	17.2	17.2	16.0	-1.2
216		27	28	28	28.7	28.0	26.4	-2.3
203	KABC-II NVI	59	55	70	80.8	67.9	80.5	-0.3
204		60	66	61	33.9	36.3	32∙5	-1.4
213		65	82	93	34.6	42.3	46.5	11.9
mean					36.1	35.1	36.1	-1.2
SEM					7.1	5.8	7.4	2.8

*na* not available

^a^Baseline DQ not available; 13-week DQ is shown

^b^52-week DQ not measured; difference computed from 26-week score

**Table 5:** Change from Baseline of DQ and AES using VABS-II at week 52

domain	DQ		AES (months)	
	baseline	52 wk-baseline	baseline	52 wk-baseline
Overall	44.4 ± 6.6	+0.6 ± 5.3	37.4 ± 10.6	+12.2 ± 8.0
Communication	42.5 ± 7.7	-1.8 ± 3.8	38.3 ± 15.0	+7.9 ± 5.1
Daily Living Skills	46.7 ± 8.4	-2.5 ± 6.0	38.2 ± 9.3	+7.4 ± 4.5
Socialization	38.7 ± 5.8	+9.5 ± 9.0	32.1 ± 8.8	+21.3 ± 16.5
Motor Ability	43.5 ± 8.2	+1.6 ± 4.6	31.6 ± 6.2	+8.4 ± 3.7

Mean ± SEM (*N*=11)

**Table 6:** Change from Baseline of DQ and AES using non-cognitive domains of BSID-III at week 52

domain	DQ		AES (months)	
	baseline	52 wk-baseline	baseline	52 wk-baseline
Receptive communication	13.8 ± 3.7	+1.5 ± 5.2	7.8 ± 2.4	+3.2 ± 2.4
Expressive communication	17.6 ± 4.0	+1.6 ± 3.1	9.3 ± 2.2	+4.3 ± 1.3
Fine motor	34.7 ± 7.7	-5.1 ± 5.1	19.8 ± 4.5	+0.1 ± 3.3
Gross motor	25.6 ± 6.2	-2.3 ± 2.0	13.4 ± 1.8	+1.8 ± 1.1

Mean ± SEM (*N*=8)

**Fig. 2:**
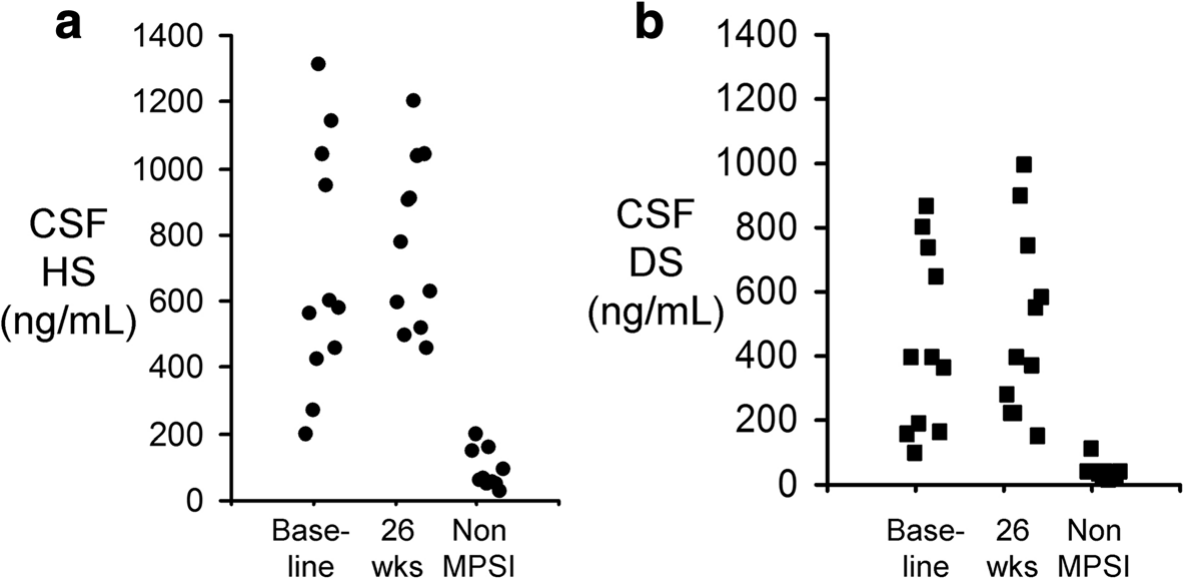
Concentration of heparan sulfate (HS, panel **a**) and dermatan sulfate (DS, panel **b**) in CSF of Stage 2 patients at baseline and at 26 weeks (wks) of treatment in comparison to HS and DS levels in CSF of 10 non-MPSI human pediatric subjects. The mean ± SE values for HS in CSF are 685 ± 112, 779 ± 78, and 91 ± 18 ng/mL for the MPSI patients at baseline, for the MPSI patients at 26 weeks, and for the pediatric controls, respectively. The mean ± SE values for DS in CSF are 436 ± 85, 490 ± 87, and 38 ± 8 ng/mL for the MPSI patients at baseline, for the MPSI patients at 26 weeks, and for the pediatric controls, respectively

The total grey matter volume at baseline was 692,631 ± 34,396 mm^3^, and the grey matter volume was stabilized over 52 weeks of treatment as the mean difference between the 52-week and baseline volumes was +15,071 ± 11,052 mm^3^ (Table 7). CSF pressure at baseline and at 26 weeks was 27.0 ± 3.5 and 33.0 ± 3.5 cm water, respectively (mean±SE). The CSF concentrations of HS and DS are shown in Fig. 2a and b, respectively, for the Stage 2 patients at baseline and at 26 weeks, along with HS and DS values for control pediatric subjects. The CSF HS in the MPSI patients at baseline, 685 ± 112 ng/mL (SEM), is 7-fold elevated above the CSF HS value for non-MPSI human subjects, 91 ± 18 (SEM) (Fig. 2a). The CSF HS concentrations at baseline and at 26 weeks of treatment, 779 ± 78 ng/mL, are not significantly different (Fig. 2a). The CSF DS in the MPSI patients at baseline, 436 ± 85 ng/mL (SEM), is 11-fold elevated above the CSF DS value for non-MPSI human subjects, 38 ± 8 (SEM) (Fig. 2b). The CSF DS concentrations at baseline and at 26 weeks of treatment, 490 ± 87 ng/mL, are not significantly different (Fig. 2b).

**Table 7:** Change from baseline in total grey matter volume at week 52

patient	age	Total grey matter volume (mm^3^)			
		BL	26 wk	52 wk	52wk-BL
202	9.0	797,018	792,018	788,648	-8,370
203	6.1	747,735	768,848	nd	+21,113^a^
204	14.8	517,491	604,125	616,132	+98,641^b^
205	5.0	710,691	759,444	749,177	+38,486
206	3.1	621,494	673,817	703,973	+82,478
207	5.9	607,678	590,810	598,460	-9,218
211	2.5	589,439	np^c^	np^c^	
213	15.7	874,710	885,006	863,862	-10,848
214	2.0	744,555	764,571	780,779	+36,224
215	12.1	668,638	663,441	676,069	+7,431
216	7.8	739,492	708,228	717,836	-21,656
mean		692,631			+15,071
SEM		34,396			11,052

*BL* baseline, *nd* volumetrics analysis could not be performed

^a^52-week volumetrics not available; difference computed from 26-week volume

^b^Patient with braces; volumes not used to compute mean difference at 52 weeks

^c^np=not performed; volumetric analysis could not be completed

**Fig. 3:**
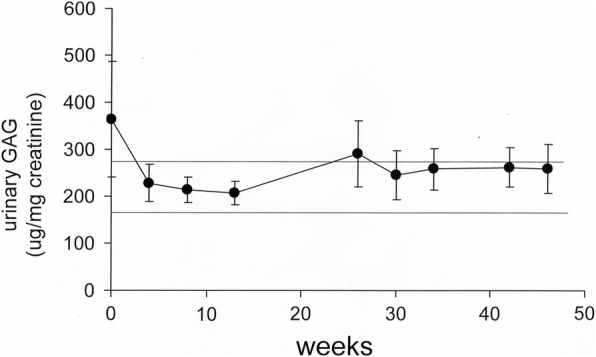
Urinary GAGs (Mean±SEM) are plotted vs weeks of treatment. The horizontal lines are the mean±SEM of urinary GAGs in 12 MPSI children after 12 months of laronidase therapy, with a range of 177-269 ug GAG/mg creatinine [4]

Signs of somatic function were stable when subjects were transferred from laronidase ERT to valanafusp alpha ERT, as shown by the urinary GAG levels (Fig. 3). Urinary GAG levels were maintained throughout the 12-month study and averaged 246 ± 14 ug/mg creatinine (mean± SEM) during weeks 4 to 46 of the study in all 11 subjects (Fig. 3). The urinary GAG in the 9 subjects that had been on laronidase ERT was 202 ± 31 ug/mg creatinine (mean ± SEM) at screening, whereas the urinary GAG at screening of the 2 naïve 2-year-olds was 744 and 1440 ug/mg creatinine. Somatic improvement in patients previously on laronidase ERT was examined with the measurement of liver and spleen volumes by MRI. Liver and spleen volumes at either baseline or at 52 weeks were normalized for body weight (BW) at baseline or 52 weeks, and reported as either total organ volume (mL) or as a percent of BW, or %BW (Table 8). The liver volume, expressed as %BW, was reduced significantly (*P*<0.0005) 23% over 52 weeks, from 3.15 ±0.33 %BW, at baseline, to 2.41 ± 0.21 %BW at 52 weeks (Table 8). If the 2 patients (211, 214) who had not previously been on ERT are excluded, the liver volume, expressed as %BW, is reduced 22% at 52 weeks. The spleen volume, expressed as %BW was reduced significantly (*P*<0.005) 26% over 52 weeks, from 0.68 ± 0.07 %BW, at baseline to 0.50 ± 0∙05 %BW, at 52 weeks (Table 8). If the 2 patients (211, 214) who had not previously been on ERT are excluded, the spleen volume, expressed as %BW, is reduced 23% at 52 weeks. Left shoulder flexion was significantly improved from 90.2 ± 2.8 degrees (SEM) at baseline to 99.8 ± 3.6 degrees at 26 weeks (*P*<0.01); right shoulder flexion was significantly improved from 87.4 ± 3.4 degrees at baseline to 99.2 ± 3.9 degrees at 26 weeks (*P*<0.01), respectively. Left shoulder extension was significantly improved from 79.5 ± 4.7 degrees at baseline to 87.6 ± 4.4 degrees at 26 weeks (*P*<0.05); right shoulder extension was significantly improved from 77.5 ± 5.1 degrees at baseline to 89.0 ± 4.7 degrees at 26 weeks (*P*<0.01), respectively. The mean increase in left and right shoulder flexion, and left and right shoulder extension is 10.7 ± 2.7 and 9.8 ± 3.5 degrees (SEM), respectively.

**Table 8:** Change from baseline of liver and spleen volumes at Week 52

patient	liver				spleen			
	baseline		52 weeks		baseline		52 weeks	
	mL	%BW	mL	%BW	mL	%BW	mL	%BW
202	445.7	2.27	404.4	1.96	76.3	0.39	87.8	0.43
203	546.1	2.86	518.7	2.44	108.9	0.57	107.3	0.50
204	930.0	2.51	800.9	1.75	149.1	0.40	107.6	0.24
205	691.1	2.80	548.8	1.78	174.0	0.70	107.1	0.35
206	570.2	4.92	486.2	3.33	87.7	0.76	75.5	0.52
207	529.3	2.82	483.7	2.27	209.7	1.12	181.1	0.85
211	528.1	4.63	434.2^a^	3.37	88.0	0.77	58.1^a^	0.45
213	1273.5	3.31	1198.4	2.65	210.7	0.55	247.1	0.55
214	601.5	4.63	530.6	3.38	139.0	1.07	118.4	0.75
215	702.6	1.77	765.7	1.50	202.9	0.51	202.5	0.40
216	578.6	2.19	642.5	2.09	168.2	0.64	156.4	0.51
mean	672.4	3.15^b^	619.5	2.41^b^	146.8	0.68^c^	131.7	0.50^c^
SEM	71.8	0.33	69.8	0.21	15.4	0.07	17.6	0.05

^a^52-week MRI could not be performed; difference computed from 26-week volume

^b^Liver %BW at 52 weeks is significantly reduced compared to %BW at baseline (*P*<0.0005)

^c^Spleen %BW at 52 weeks is significantly reduced compared to %BW at baseline (*P*<0.005)

The titer of ADAs, which detects antibodies against both laronidase and valanafusp alpha, over the course of 52 weeks is shown in Table 9. There was little correlation between clinical IRRs and ADA titer. Patient 202 presented with a single IRR at week 32, but had a low ADA titer. Patient 207, whom had previously been on ERT for 3 years, and who had a very high ADA titer at baseline, exhibited a single IRR at week 24. Patient 213, who had been on ERT for 4 years, and presented with a high ADA titer at baseline, exhibited a single IRR at week 2. Patient 214, an ERT-naïve 2-year-old, developed 6 IRRs between weeks 2-11 of treatment, and then developed clinical tolerance to valanafusp alpha; however, the ADA titer in this patient reached maximal levels by week 26 of treatment. Nine of the 11 patients (82%) had ADA titers below 10^5^ throughout the 52 weeks of treatment (Table 9).

**Table 9:** Valanafusp alpha anti-drug antibody (ADA) titers

	Week	Patient number										
		202	203	204	205	206	207	211	213	214	215	216
ADA^a^ titer	0	0	0	0	0	450	36,450	0	36,450	0	1,350	1,350
	2	0	0	0	0	450	109,350	0	4,050	0	1,350	4,050
	4	0	4,050	0	0	450	328,050	1,350	12,150	4,050	150	4,050
	8	50	0	0	150	1,350	328,050	0	109,350	12,150	150	1,350
	12	0	0	0	50	1,350	328,050	450	36,450	36,450	150	12,150
	16	150	0	0	0	1,350	109,350	1,350	36,450	36,450	150	4,050
	20	150	0	0	1,350	450	109,350	1,350	36,450	109,350	12,150	12,150
	26	150	0	0	50	4,050	109,350	12,150	36,450	328,050	4,050	36,450
	39	50	150	0	50	50	109,350	36,450	36,450	328,050	4,050	12,150
	52	50	450	50	450	1,350	109,350	12,150	35,450	328,050	4,050	36,450
ERT^b^		8	2	5	1	2	3	0	4	0	4	1
IRR^c^		1	0	0	0	0	1	1	1	6	0	0

^a^ADA titer is dilution of serum giving ELISA response above background

^b^ERT is the number of years on laronidase treatment prior to enrollment

^c^IRR is the number of infusion related reactions over 52-week study

## Discussion

MPSI presents with different clinical severity and MPSIH (Hurler syndrome) is the most severe form. If untreated, patients suffer from a severe neurocognitive decline starting at an age of one year and the median DQ declines 14-17 points per year [16]. As laronidase does not cross the BBB [5], the only therapeutic option to prevent further neurocognitive decline is HSCT. HSCT is considered to prevent loss of DQ if administered to children at a young age, usually before age of 16 months [6]. None of the severe MPSI patients included in this study underwent a successful HSCT and the severity of the disease would predict further neurocognitive decline. The goal of this study was to investigate the safety of weekly infusions of valanafusp alpha in patients with severe MPSI and its effect on the somatic and neurocognitive disease stabilization. The results of this clinical study are consistent with the following conclusions. First, valanafusp alpha has an acceptable safety profile (Table 3), with drug-related adverse events limited to transient hypoglycemia (6.4%) and infusion related reactions (1.7%). Second, chronic weekly IV infusions of valanafusp alpha stabilize CNS function in severe forms of MPSI as judged by cognitive testing (Tables 4, 5 and 6), and total grey matter volume (Table 7). Third, valanafusp alpha also stabilizes signs of somatic function as shown by further reductions in liver and spleen volume (Table 8), further improvement in shoulder range of motion (Results), and stabilized urinary GAG levels (Fig. 3).

In the course of this 52-week study, over 500 IV infusions of valanafusp alpha were administered to 11 pediatric subjects with severe MPSI, and the overall incidence of transient hypoglycemia was 6.4%. However, 67% of the hypoglycemic episodes were observed following the infusion of the high dose, 6 mg/kg, of valanafusp alpha. The higher incidence of hypoglycemia at the 6 mg/kg dose is predicted from the pharmacokinetics of valanafusp alpha clearance from plasma, which is linear over the dose range of 0.3 to 3 mg/kg, and is 4-fold faster in children as compared to adults [11]. In children, the plasma area under the concentration curve (AUC) increases 6-fold when the infusion dose is doubled from 3 to 6 mg/kg [11]. This non-linearity at the 6 mg/kg dose is attributed to partial saturation and/or down-regulation of the peripheral M6PR at the high dose of fusion protein, and produces very high plasma concentrations of valanafusp alpha [11]. If hypoglycemia at the 6 mg/kg dose is excluded, the incidence of hypoglycemia in the 1-3 mg/kg infusions was 2.1%. Of the 12 episodes of transient hypoglycemia at the 1-3 mg/kg dose, all but 1 episode was grade 1 (glucose 50-59 mg%). The hypoglycemia occurs during the 3-4 hour infusion of valanafusp alpha, and the plasma glucose is normal for up to 24 hours after the end of the infusion even at the 6 mg/kg dose [11]. Hyperglycemia was not observed over the 52 weeks of treatment, which is consistent with results from the primate pharmacology study. Preclinical studies in Rhesus monkeys demonstrated that there is no change in glycemic control following 6 months of treatment of Rhesus monkeys with valanafusp alpha [17].

The overall incidence of IRRs with valanafusp alpha was 1.7%, and over 60% of these IRRs were observed in a single patient (Results). No anaphylactic reactions were observed. The rate of IRR observed with laronidase was 52% in a meta-analysis of 73 patients with MPSI [18]. The development of valanafusp alpha ADAs is comparable to the formation of ADAs with laronidase ERT [18]. The number of MPSI patients on laronidase ERT that develop ADA titers <100, <6,400, <51,000, and >51,000 is 3, 22, 40, and 34% [18]. In the present study of 11 subjects, the incidence of ADA titers at these levels is 18, 36, 27, and 18%, respectively (Table 9). Some ADAs may be neutralizing antibodies (NAb) and inhibit the uptake of valanafusp alpha into peripheral tissues via the M6PR [18]. The likelihood of NAb formation is increased when the ADA titer is >10^4^-10^5^, as ADA titers at this level correlates with elevations in urinary GAGs [4, 18]. In the present study, urinary GAGs remained low throughout the 52 weeks of treatment (Fig. 3), with 2 exceptions. Both patient 211 and 214, the ERT-naïve 2-year-old subjects, exhibited an increase in urinary GAG at 26 weeks to 772 and 696 ug GAG/mg creatinine, respectively. However, the urinary GAG returned to <400 ug/mg creatinine in both naïve patients during the latter 6 months of treatment.

Valanafusp alpha is a BBB-penetrating form of IDUA, and is designed to deliver the IDUA enzyme to brain cells to reverse the accumulation of heparan sulfate (HS) GAGs and lysosomal inclusions. The primary therapeutic goal of valanafusp alpha therapy is stabilization of cognitive decline in severe MPSI. The decline in cognitive function in MPSI may be a 2-step process, where the first pathologic event is the formation of intra-neuronal lysosomal inclusion bodies, which is then followed by a secondary neuropathology leading to dystrophic neurites and cognitive decline. However, the reversal of cognitive decline, resulting in an actual increase in cognitive function, requires the subsequent repair of dystrophic neurites, and valanafusp alpha is not expected to directly intervene in such neural repair. In the present study, a significant increase in DQ in this severely impaired cohort of MPSI subjects was not observed. However, the DQ was stabilized and the mean difference between the 52 week DQ and the baseline DQ was -1.2 ± 2.8 points (Table 4). MPSI patients have a favorable outcome with respect to cognitive function if the child is transplanted early before the age of 16 months when the DQ is still >85 [6]. In the present study, the mean age and DQ of the MPSI subjects was 7.7 ± 1.4 years, and 36.1 ± 7.1, respectively (mean±SEM). The secondary measures of DQ were also stabilized by valanafusp alpha therapy, including the VABS-II (Table 5) and other domains of the BSID-III (Table 6). The age equivalent scores (AES) increased over the course of the 52 week study across multiple domains of cognitive testing (Table 4, 5 and 6), which contrasts with the untreated MPSIH patient, where the AES declines after the age of 3 years [16]. The volume of the total grey matter was stabilized by valanafusp alpha treatment (Table 7), and the grey matter volume increased 20-82 cm^3^ in younger subjects 2-6 years of age (Table 7). There is evidence for a correlation between preservation of grey matter volume and cognitive function in MPS [19, 20].

The GAGs elevated in MPSI are heparan sulfate (HS) and dermatan sulfate (DS), and HS and DS, as measured by LC-MS, are elevated in CSF in the MPSI subjects tested in this study (Fig. 2). Valanafusp alpha treatment for 6 months did not result in a significant change in HS or DS in lumbar CSF (Fig. 2). However, HSCT similarly does not cause a reduction in HS in CSF in patients with MPSI [21]. In patients with MPSIIIB treated with intra-cerebral gene therapy, despite an apparent improvement in cognitive function, the HS level in CSF actually increases after treatment [22]. Cognition in MPS may be related to GAG concentrations in the parenchyma of brain, but lumbar CSF GAGs may not be a surrogate marker of GAGs in brain parenchyma. The intrathecal injection of recombinant iduronate 2-sulfatase into lumbar CSF in MPSII [23], or the intrathecal injection of recombinant sulfamidase into lumbar CSF in MPSIIIA [24], results in a reduction in lumbar CSF GAG concentrations, but this reduction in CSF GAG is not associated with any improvement in cognitive function. Animal studies suggest that GAGs in CSF may be derived primarily from the dura mater of the spinal cord [25]. It may be necessary to directly sample brain parenchymal tissue to accurately assess GAG levels in the CNS. When brain tissue is directly measured for GAGs in mouse models of MPS, the intravenous administration of BBB-penetrating IgG-enzyme fusion proteins results in a >70% reduction in brain parenchymal lysomal inclusion bodies in the MPSI mouse [10], or a >70% reduction in brain parenchymal HS in the MPSIIIA mouse [15].

Valanafusp alpha has dual receptor targeting properties, where the HIRMAb domain of the fusion protein targets the insulin receptor, and the IDUA domain targets the M6P receptor. The M6PR plays the dominant role in the clearance of valanafusp alpha from plasma, as the plasma clearance of valanafusp alpha in primates is 250-fold faster than is the clearance of the HIRMAb alone at an infusion dose of 3 mg/kg [26]. Owing to the primary role played by the M6PR in the peripheral clearance of valanafusp alpha, the biodistribution of valanafusp alpha and laronidase is comparable in primates [5]. The rate of plasma clearance of valanafusp alpha and laronidase in pediatric subjects with MPSI is also comparable [11]. The similarity in peripheral clearance of valanafusp alpha and laronidase underlies the continued somatic stabilization in MPSI with valanafusp alpha treatment. The somatic disease stabilization by valanafusp alpha is reflected in the maintenance of urinary GAGs at the same level as that maintained by laronidase (Fig. 3). Laronidase treatment reduces liver and spleen volumes. However, this reduction is maximal by 6 months of treatment and continued treatment, or increasing the dose of laronidase, does not result in further decreases in liver volume [27]. In the present study, a year of treatment with valanafusp alpha resulted in further reductions in liver and spleen volume, as a percent of body weight, of 23% and 26% (mean±SEM), respectively (Table 8). Valanafusp alpha treatment also resulted in a 10.7 ± 2.7 degree and 9.8 ± 3.5 degree improvement in shoulder flexion and extension, respectively (Results), which is a clinically significant improvement of joint mobility [28].

## Conclusion

We conclude the present 12 month study demonstrates that valanafusp alpha has a favorable safety profile, chronic weekly IV infusions of valanafusp alpha stabilize CNS function in severe forms of MPSI, and valanafusp alpha stabilizes signs of somatic function. A larger, controlled clinical trial is warranted testing cognitive and somatic function in MPSI with up to 2 years of treatment with valanafusp alpha at the dose of 3 mg/kg.

## Abbreviations

ADA: Anti-drug antibody
AES: Age equivalent score
BBB: Blood-brain barrier
BL: Baseline
BSID-III: Bayley Scales of Infant and Toddler Development, Third Edition
BW: Body weight
CNS: Central nervous system
CSF: Cerebrospinal fluid
DQ: Development quotient
DS: Dermatan sulfate
Dx: Age of diagnosis
ERT: Enzyme replacement therapy
GAG: Glycosaminoglycan
H: Hurler syndrome
HIR: Human insulin receptor
HS: Heparan sulfate
H-S: Hurler-Scheie syndrome
HSCT: hematopoietic stem cell transplant
ID: Infusion dose
IDUA: Iduronidase
IRR: Infusion related reaction
IV: Intravenous
KABC-II: Kaufman Assessment Battery for Children, Second Edition
M6P: Mannose 6-phosphate
M6PR: M6P receptor
MAb: Monoclonal antibody
MPS: Mucopolysaccharidosis
MPSI: MPS Type I
MPSIH: Hurler syndrome form of MPSI
MPSII: MPS Type II
MPSIIIA: MPS Type IIIA
NAb: Neutralizing antibody
NVI: Non-verbal index
PK: Pharmacokinetics
rINN: International non-proprietary
ROM: Range of motion
SEM: Standard error of the mean
uGAG: Urinary GAG
URI: Upper respiratory infection
VABS-II: Vineland Adaptive Behavior Scales, Second Edition
